# Supplementary material for: Oxygen-Plasma-Induced Hetero-Interface NiFe2O4/NiMoO4 Catalyst for Enhanced Electrochemical Oxygen Evolution
Source: Materials (Basel). 2022 May 20;15(10):3688. doi: 10.3390/ma15103688 (PMC9146484; doi:10.3390/ma15103688)
Supplement: Supplementary file 1 [file materials-15-03688-s001.zip › materials-1718394-supplementary.pdf]

Oxygen plasma induced hetero-interface $NiFe_2O_4/NiMoO_4$ catalyst for enhanced electrochemical oxygen evolution

Nuo Xu [1, ‡], Wei Peng [2, ‡], Lei Lv [2], Peng Xu [2], Chenxu Wang [1], Jiantao Li [3], Wen Luo [1,2,*], Liang Zhou [2]

[1] Department of Physics, School of Science, Wuhan University of Technology, Wuhan 430070, P.R. China

[2] State Key Laboratory of Advanced Technology for Materials Synthesis and Processing, Wuhan University of Technology, Wuhan 430070, P.R. China

[3] Chemical Sciences and Engineering Division, Argonne National Laboratory, Illinois 60439, USA

E-mail: luowen_1991@whut.edu.cn

EXPERIMENTAL SECTION

Materials. Nickel(II) chloride hexahydrate ($NiCl_2 \cdot 6H_2O$, ⩾ 98%) and sodium molybdate dihydrate ($Na_2MoO_4 \cdot 2H_2O$, ⩾99.0%) were purchased from Sinopharm Chemical Reagent Co., Ltd. Potassium hydroxide (KOH) and potassium hexacyanoferrate(III) ($K_3[Fe(CN)_6]$, ⩾99%) were purchased from Aladdin Reagent (Shanghai) Co., Ltd.

Pretreatment of Ni Foam. A Ni foam (1.0 × 4.0 cm^2) was rinsed with 1.0 M HCl, acetone, and ethanol under sonication for 15 min, respectively, then dried in vacuum oven at 60 °C for 6 h.

Synthesis of $NiMoO_4$. $NiCl_2 \cdot 6H_2O$ (0.175 g) and $Na_2MoO_4 \cdot 2H_2O$ (0.145 g) were dissolved in deionized water (DI water, 10 mL) respectively, A latter solution is slowly added to the former under stirring and then kept stirring for 30 min. This mixed solution was then transferred to a 25 mL Teflon-lined autoclave. A pretreated Ni foam was then put into the autoclave containing the above mixed solution, followed by heating at 160 °C for 4 h. After cooling to room temperature naturally, the sample was taken out, rinsed with DI water and ethanol, and dried in the vacuum oven at 60 °C for 12 h.

Synthesis of NiFe PBA/$NiMoO_4$. 0.8 mg of $K_3[Fe(CN)_6]$ was dissolved in a 10 mL mixed solution of 7.5ml ethanol and 2.5ml H_2O. The as-prepared $NiMoO_4$ on Ni foam were placed into above solutions and kept for 3 h under stirring. The samples were taken out, rinsed with ethanol, and dried in a vacuum oven at 60 °C for 12 h.

Preparation of $NiFe_2O_4$/$NiMoO_4$. The as prepared NiFe PBA/$NiMoO_4$ were placed into the chamber for treatment which was conducted in the oxygen plasma environment

for 1 h. The electrical power of the plasma was set at 90 W (CTP-2000K PLASMA GENERATOR). The as prepared $NiMoO_4$ on Ni foam were also treated as a comparison sample under same treatment condition (denoted as $NiMoO_4$ O_2-Pl).

Characterization. Scanning electron microscopic (SEM) images were collected using a JEOL JSM-7100F. Transmission electron microscope (TEM), high resolution transmission electron microscope (HRTEM) and energy dispersive spectroscopy (EDS) were using Talos F200S. The chemical forming, states and atomic structure information were analyzed by XPS (Thermo-Fisher Scientific-EDCALAB 250Xi) and XRD (D8 Advance X-ray diffractometer with a Cu Kα radiation source). Raman spectroscopy was obtained by DXR, Thermo-Fisher Scientific, with 532 nm excitation from an argon-ion laser), and Fourier-transform infrared (FT-IR) spectrum was obtained by Nexus, Thermo Nicolet.

Electrochemical Measurements. For determining the electrochemical properties of all samples, all the tests are performed on the CHI 760e electrochemical workstation in 1 M KOH solution as the electrolyte solution. Cyclic voltammetry (CV), linear sweep voltammetry (LSV), electrochemical impedance spectroscopy (EIS) and chronopotentiometry were adopted to test the electrochemical performance through a three-electrode system, and the prepared catalyst is directly adopted as the working electrode, mercuric oxide electrode as the reference electrode, graphite as the counter electrode, respectively. For standardization, all tested voltages are converted to a standard reversible hydrogen electrode (RHE), based on the following formula:

$E_{RHE} = 0.098 + 0.059 \times pH + E_{Hg/HgO}$

The catalyst was activated by CVs at a scanning rate of 5 mV s^{-1} until it reached a stable state. LSV curves are obtained at a scan rate of 5 mV s^{-1} with *iR* corrected. The Tafel slope is obtained by plotting the LSV curve of the potential relative to log (current density). The Tafel slope can be used to more intuitively analyze the relationship between overpotential and current density, providing more effective and direct kinetic information for catalyst reaction mechanism. EIS was tested at 1.47 V against to RHE from 0.01Hz to 100 kHz. Chronopotentiometry was performed at a current density of 50 mA cm^{-2} to assess its long-term stability. The electrochemical active surface area (ECSA) is indicated by an electrochemical double-layer capacitance (C_{dl}) derived from CVs at a potential of 0.05-0.15 V relative to the Hg/HgO with a scan rate of 20, 40, 60, 80 and 100 mV s^{-1}, respectively.

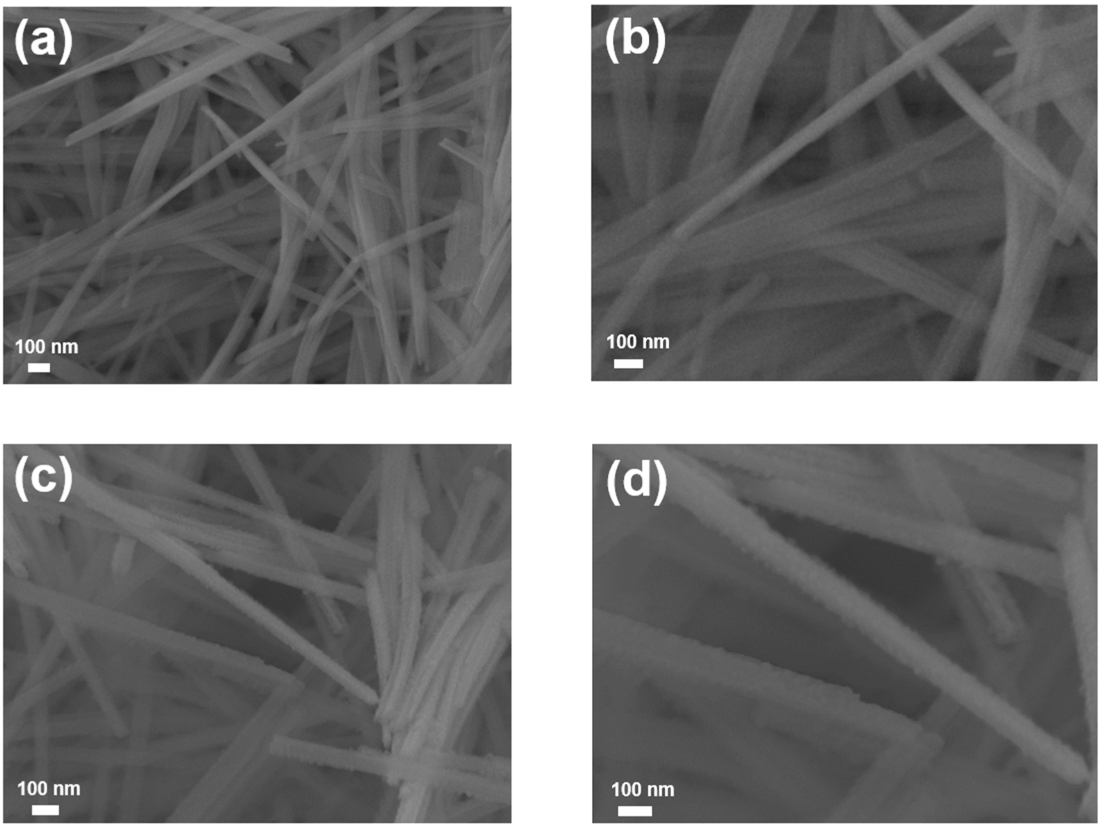

Figure S1. SEM images (a, b) and (c, d) of $NiMoO_4$ and NiFe PBA/$NiMoO_4$, respectively.

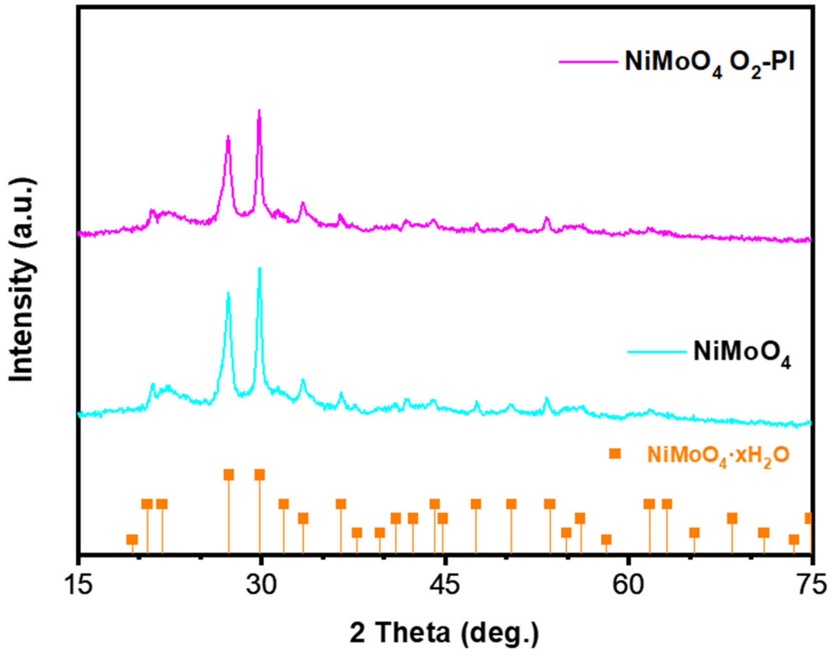

Figure S2. XRD patterns of NiFe PBA/$NiMoO_4$ and $NiFe_2O_4$/$NiMoO_4$.

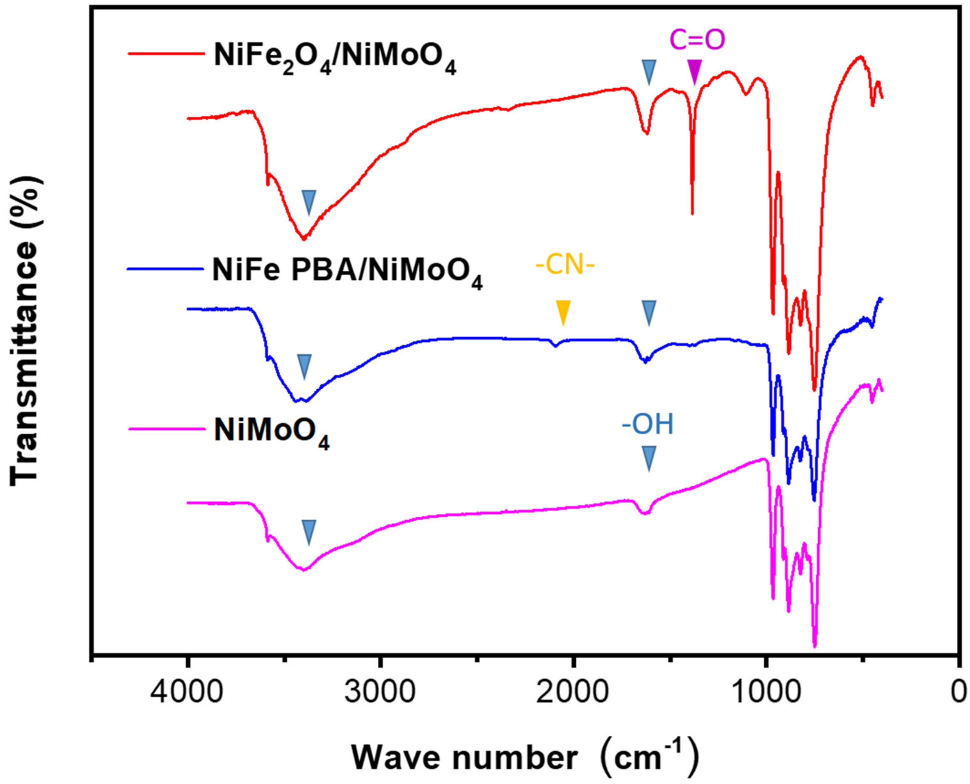

Figure S3. FT-IR spectra of $NiMoO_4$, NiFe PBA/$NiMoO_4$ and $NiFe_2O_4$/$NiMoO_4$

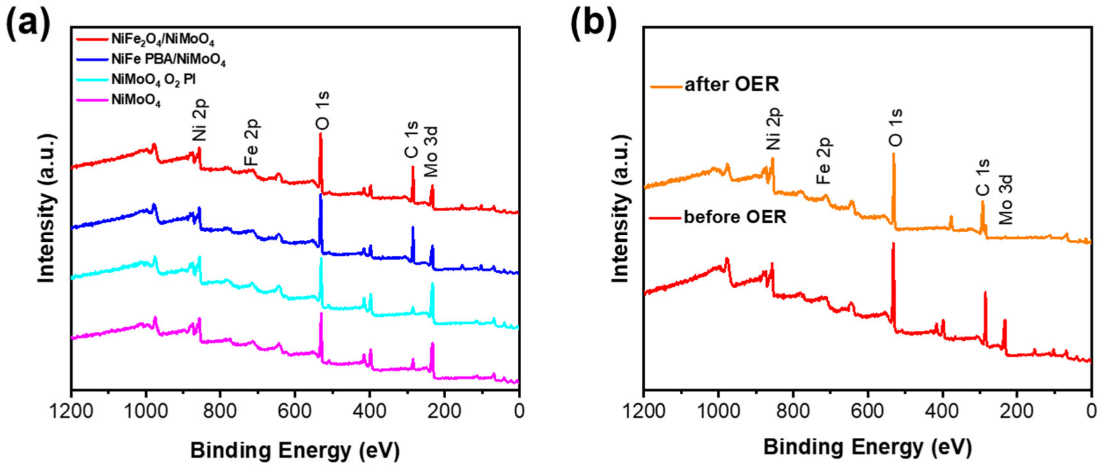

Figure S4. XPS spectrum of (a) $NiMoO_4$, $NiMoO_4$ O_2-Pl, NiFe PBA/$NiMoO_4$, $NiFe_2O_4$/$NiMoO_4$, and of (b) $NiFe_2O_4$/$NiMoO_4$ before OER and after OER.

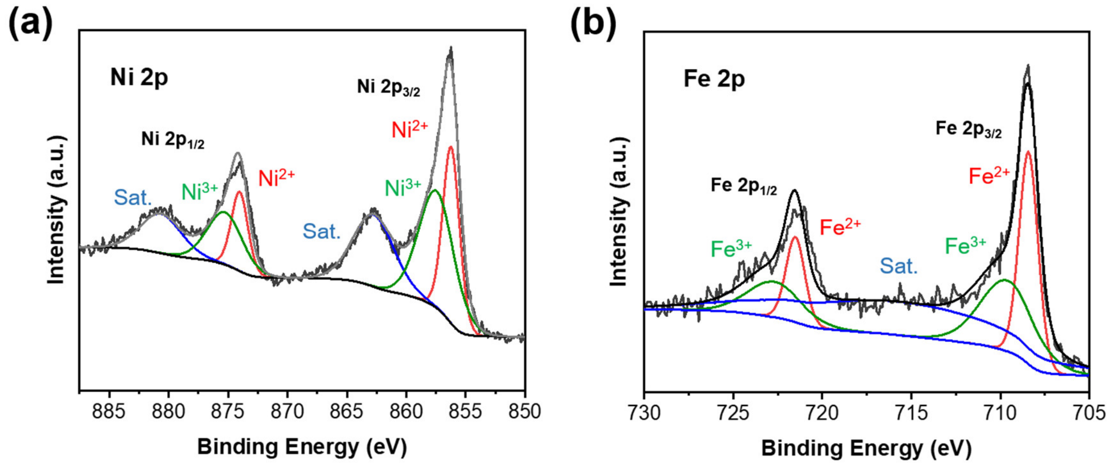

Figure S5. XPS (a) Ni 2p and (b) Fe 2p spectra of NiFe PBA/$NiMoO_4$.

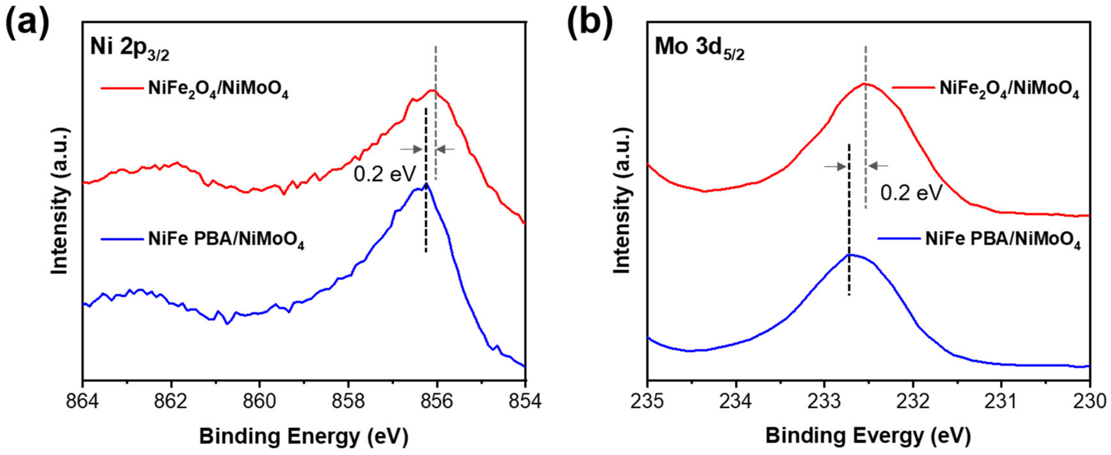

Figure S6. XPS analysis of NiFe PBA/$NiMoO_4$ and $NiFe_2O_4$/$NiMoO_4$. The core level spectra of (a) Ni $2p_{3/2}$ and (b) Mo 3d.

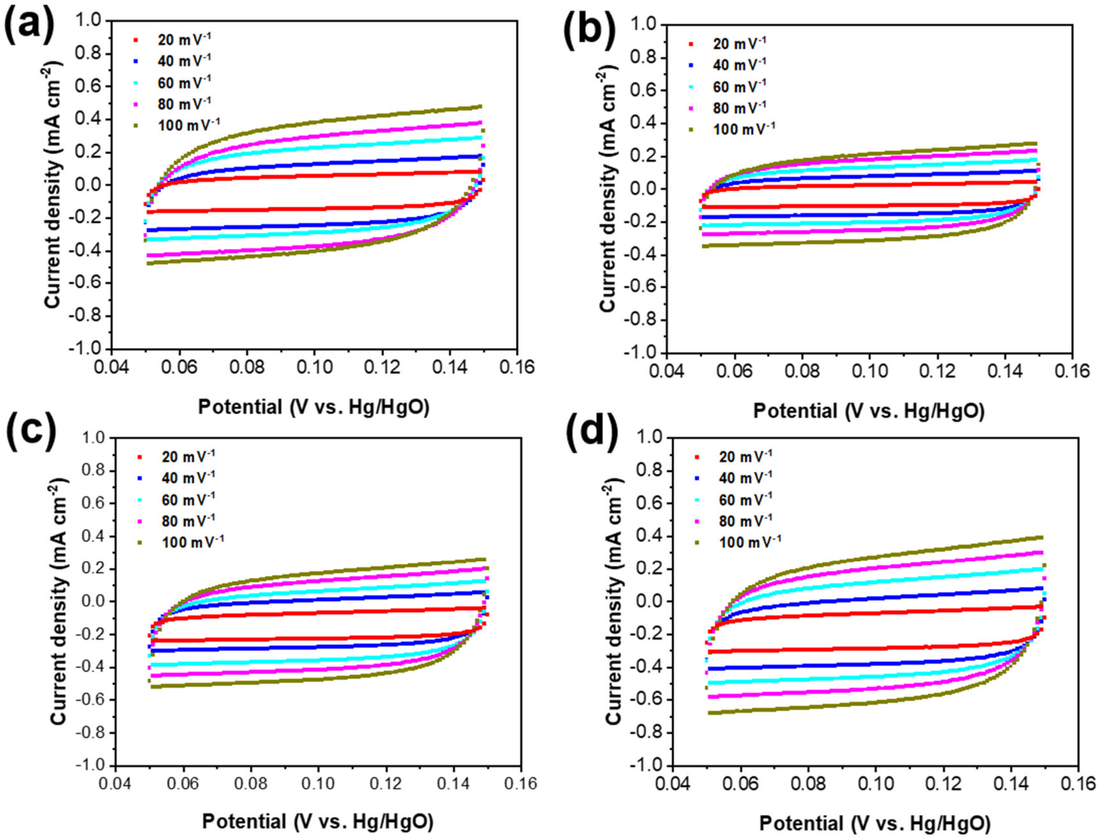

Figure S7. Cyclic voltammograms in a capacitive current region at various scan rates from 20 to 100 mV s^{-1}. (a) $NiMoO_4$, (b) $NiMoO_4$ O_2-Pl, (c) NiFe PBA/$NiMoO_4$, (d) $NiFe_2O_4$/$NiMoO_4$.

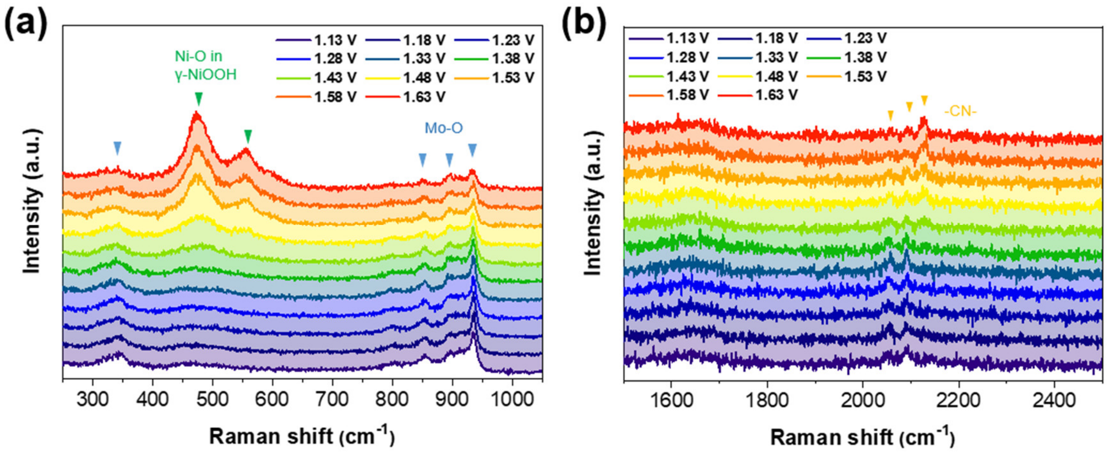

Figure S8. *In situ* Raman spectra of $NiFe_2O_4/NiMoO_4$for activation from 1.18 V to 1.63 V (a) in a region from 250 cm^{-1} to 1050 cm^{-1} and (b) in a region from 1500 cm^{-1} to 2500 cm^{-1}.

Table S1. Chemical composition of $NiFe_2O_4/NiMoO_4$ based on EDS.

Element	Atomic Fraction (%)	Mass Fraction (%)
O	75.26	39.61
Fe	1.18	2.17
Ni	13.17	25.44
Mo	10.39	32.78
